# High-sensitivity modified Glasgow prognostic score (HS-mGPS) Is superior to the mGPS in esophageal cancer patients treated with chemoradiotherapy

**DOI:** 10.18632/oncotarget.21734

**Published:** 2017-10-11

**Authors:** Peng Chen, Min Fang, Qiuyan Wan, Xuebang Zhang, Tao Song, Shixiu Wu

**Affiliations:** ^1^ Department of Radiation Oncology, Hangzhou Cancer Hospital, Hangzhou 310000, Zhejiang, P. R. China; ^2^ Department of Radiation Oncology, Zhejiang Provincial People’s Hospital, People’s Hospital of Hangzhou Medical College, Hangzhou 310000, Zhejiang, P. R. China; ^3^ Department of Gynecologic Oncology, Jiangxi Cancer Hospital, Nanchang 330029, Jiangxi, P. R. China; ^4^ Department of Radiation Oncology, The First Affiliated Hospital of Wenzhou Medical University, Wenzhou 325000, Zhejiang, P. R. China

**Keywords:** esophageal squamous cell carcinoma, Glasgow prognostic score, prognostic factor, treatment response, survival

## Abstract

The present study compared the prognostic value of the modified Glasgow prognostic score (mGPS) and high-sensitivity mGPS (HS-mGPS) in unresectable locally advanced esophageal squamous cell carcimona (LAESCC) patients treated with concurrent chemoradiotherapy (CCRT). The baseline data of 163 eligible patients were retrospectively collected. Patients with a C-reactive protein (CRP) ≤ 10 mg/l and albumin ≥ 35 g/l were allocated to mGPS-0 group. Patients with only elevated CRP (> 10 mg/l) were assigned to mGPS-1 group. Patients who had both elevated CRP (> 10 mg/l) and hypoalbuminurea (< 35 g/l) were assigned to mGPS-2 group. The HS-mGPS was calculated based on cutoff values of 3mg/l for CRP and the same value (35 g/l) for albumin. Prognostic significance for both tumor response and overall survival (OS) was analyzed by univariate and multivariate analysis. The mGPS was 0 in 95 patients, 1 in 28 patient and 2 in 40 patients. In contrast, the HS-mGPS was 0 in 66 patients, 1 in 47 patients and 2 in 50 patients. In multivariate analysis, the HS-mGPS was the only positive factor for tumor response (*P =* 0.015). Both the mGPS (*P* < 0.001) and HS-mGPS (*P* < 0.001) were good prognostic predictors for OS. However, the HS-mGPS was found to be a superior prognostic predictor compared to the mGPS in a multivariate analysis (*P =* 0.006). In conclusion, the pretreatment HS-mGPS is a strong prognosticator superior to the mGPS for both tumor response and OS in LAESCC patients who received CCRT.

## INTRODUCTION

Esophageal squamous cell carcinoma (ESCC) is a common malignancy with a high burden of morbidity and mortality in China. More than 50% ESCC patients are diagnosed at the advanced stages [1]. Concurrent chemoradiotherapy (CCRT) has been widely accepted as a valuable curative treatment option for locally advanced ESCC (LAESCC) patients who choose non-surgical management [2]. However, the long-term survival rate of LAESCC patients remains dismal with no better than 20%. Therefore, there is continuing momentum in finding effective prognostic factors that could facilitate accurate patient stratification, and further improve therapeutic outcomes.

The modified Glasgow prognostic score (mGPS) is calculated based on the serum concentrations of C-reactive protein (CRP; cutoff value: 10 mg/l) and albumin (ALB; cutoff value: 35 mg/l) levels, which focusing on systemic inflammation and nutritional status in cancer patients. It has been validated as an independent prognostic factor in various malignancies including esophageal cancer [3]. Accompany with the advancement of laboratory measurements, some experts have suggested that a lower threshold for CRP (cutoff value: 3 mg/l) may enhance the prognostic value of the mGPS in cancer patients, and a high-sensitivity mGPS (HS-mGPS) has been proposed [4–7].

To date, there is a paucity of studies in the literature clarifying the prognostic effects of mGPS and HS-mGPS in predicting treatment response and prognosis in unresectable LAESCC patients who received CCRT. Therefore, the purposes of this study were: (1) to determine the impact of mGPS and HS-mGPS on tumor response; (2) to assess the prognostic effect of mGPS and HS-mGPS on overall survival (OS); (3) to compare the prognostic efficiency of mGPS and HS-mGPS in LAESCC patients.

## RESULTS

### Patient characteristics

Of the 163 LAESCC patients, the median age at diagnosis was 57 years (range, 31–79 years), and 134 patients were male while another 29 were female. Approximately 83.4% (*n* = 136) of patients were diagnosed with stages III–IV. 70 patients had an ECOG PS score of 0–1 and 114 patients received CCRT based on TP regimen. In total, 95 patients had an mGPS of 0, 28 had an mGPS of 1, and 40 had an mGPS of 2. The mGPS was significantly correlated with N stage (*P* = 0.047), M stage (*P* < 0.001), clinical stage (*P* = 0.002), BMI (*P* = 0.024) and treatment modality (*P* = 0.040). In contrast, 66 had an HS-mGPS of 0, 47 had an HS-mGPS of 1, and 50 had an HS-mGPS of 2. The HS-mGPS was significantly correlated with N stage (*P* = 0.038), M stage (*P* < 0.001), clinical stage (*P* < 0.001), and BMI (*P* = 0.015). Similar to the mGPS, an increase in the HS-mGPS was associated with more progressive disease and pretreatment malnutrition (Table 1).

**Table 1:** Correlation between the baseline clinical characteristics and the mGPS/HS-mGPS

Factor	Total (*n*, %)	mGPS				HS-mGPS			
		mGPS 0	mGPS 1	mGPS 2	*P*-value	HS-mGPS 0	HS-mGPS 1	HS-mGPS 2	*P*-value
All	163 (100)	95	28	40		66	47	50	
Age (years)					0.209				0.106
Median (range)	57 (31–79)								
< 57	70	42	15	13		29	25	16	
≥ 57	93	53	13	27		37	22	34	
Sex					0.514^a^				0.916
Male	134	76	25	33		54	38	42	
Female	29	19	3	7		12	9	8	
ECOG PS					0.134				0.187
0–1	70	47	9	14		34	17	19	
2	93	48	19	26		32	30	31	
T stage					0.361				0.656
3	87	55	14	18		37	26	24	
4	76	40	14	22		29	21	26	
N stage					0.047				0.038
0	52	37	8	7		26	17	9	
1	111	58	20	33		40	30	41	
M stage					<0.001				<0.001
0	98	65	19	14		47	33	18	
1_a_	65	30	9	26		19	14	32	
Clinical stage (AJCC 2002)					0.002^a^				<0.001^a^
II	27	21	4	2		15	10	2	
III	71	44	15	12		32	23	16	
IV_a_	65	30	9	26		19	14	32	
Tumour length (cm)					0.456				0.527
< 5	66	40	13	13		29	20	17	
≥ 5	97	55	15	27		37	27	33	
Tumor location					0.984				0.781
Upper third	62	35	12	15		22	18	22	
Middle third	63	37	10	16		26	19	18	
Lower third	38	23	6	9		18	10	10	
Differentiation					0.932				0.965
Well	39	24	6	9		17	10	12	
Fairly	63	38	11	14		26	19	18	
Poorly	61	33	11	17		23	18	20	
Weight loss					0.569				0.162
< 10%	105	63	19	23		42	35	28	
≥ 10%	58	32	9	17		24	12	22	
Body mass index (BMI, Kg/m^2^)					0.024				0.015
BMI < 18.5	37	13	9	15		7	13	17	
18.5 ≤ BMI < 22.9	94	62	13	19		45	22	27	
BMI ≥ 23	32	20	6	6		14	12	6	
RT delivery					0.616				0.662
3D-CRT	106	59	20	27		41	33	32	
IMRT	57	36	8	13		25	14	18	
Treatment modality					0.040				0.054
TP	114	73	19	22		52	33	29	
PF	49	22	9	18		14	14	21	

Note: *n*: number of patients; mGPS: modified Glasgow Prognostic Score; HS-mGPS: High-sensitivity modified Glasgow Prognostic Score; ECOG: Eastern Cooperative Oncology Group; BMI: Body mass index; RT: radiotherapy; 3D-CRT: three-dimensional conformal radiotherapy; IMRT: intensity-modulated radiation therapy; TP: cisplatin and paclitaxel; PF: cisplatin and 5-Fu. ^a^: Mann-Whitney *U* test, others were compared with χ^2^ test.

### Predictive factors for the treatment response

The treatment response was evaluated according to RECIST. CR was observed in 34 (20.9%), PR in 45 (27.6%), SD and PD in 84 (51.5%) patients, which yielded an objective response rate (ORR) of 48.5%. Univariate analysis of predictive factors for the response to CCRT showed that mGPS (*P* = 0.005) and HS-mGPS (*P* = 0.001) were strongly correlated with a better treatment response (Table 2). Other variables associated with an ORR were the T stage (*P* = 0.014), N stage (*P* = 0.010), clinical stage (*P* = 0.003) and tumor length (*P* = 0.011). Moreover, patients who received CCRT based on TP regimen showed a better ORR than patients who received PF regimen (*P* = 0.022). Multivariate analysis identified HS-mGPS as the only independent predictive factor for ORR (*P* = 0.015) (Table 2).

**Table 2:** Univariate and multivariate analysis for tumor response

Variables	Univariate analysis		Multivariate analysis with mGPS		Multivariate analysis with HS-mGPS	
	HR (95% CI)	*P*-value	HR (95% CI)	*P*-value	HR (95% CI)	*P*-value
*Age (y)*		0.331		-		-
< 57	1.000					
≥ 57	1.361 (0.731–2.536)					
*Sex*		0.401		-		-
Male	1.000					
Female	1.417 (0.628–3.194)					
*ECOG PS*		0.331		-		-
0–1	1.000					
2	1.361 (0.731–2.536)					
*T stage*		0.014		0.130		0.111
T_3_	1.000		1.000		1.000	
T_4_	2.190 (1.168–4.105)		1.707 (0.854–3.415)		1.766 (0.878–3.549)	
*N stage*		0.010		0.255		0.294
N_0_	1.000		1.000		1.000	
N_1_	2.454 (1.244–4.840)		1.549 (0.729–3.288)		1.502 (0.703–3.209)	
*Clinical stage*		0.003		0.239		0.278
II–III	1.000		1.000		1.000	
IV_a_	2.717 (1.416–5.215)		1.588 (0.735–3.432)		1.541 (0.706–3.367)	
*Tumour length (cm)*		0.011		0.069		0.063
< 5	1.000		1.000		1.000	
≥ 5	2.288 (1.207–4.335)		1.912 (0.950–3.849)		1.949 (0.965–3.940)	
*Tumour location*				-		-
Upper	1.000					
Middle	0.851 (0.422–1.718)	0.653				
Lower	0.976 (0.435–2.193)	0.954				
*Differentiation*				-		-
Well	1.000					
Fairly	1.254 (0.562–2.798)	0.581				
Poorly	1.864 (0.827–4.202)	0.132				
*Weight loss (%)*		0.180		-		-
< 10	1.000					
≥ 10	1.558 (0.815–2.979)					
*BMI (Kg/m*^2^*)*		0.889		-		-
< 18.5	1.000					
18.5≤BMI<22.9	0.815 (0.380–1.746)	0.598				
≥ 23	1.093 (0.422–2.831)	0.855				
*RT delivery*		0.389		-		-
3D-CRT	1.000					
IMRT	1.329 (0.696–2.539)					
*Treatment modality*		0.022		0.299		0.328
TP	1.000		1.000		1.000	
PF	2.244 (1.121–4.493)		1.524 (0.688–3.374)		1.488 (0.671–3.299)	
*mGPS*		0.005		0.077		-
0	1.000		1.000			
1	1.094 (0.469–2.548)		0.992 (0.405–2.434)			
2	3.327 (1.490–7.430)		2.306 (0.978–5.442)			
*HS-mGPS*		0.001		-		0.015
0	1.000				1.000	
1	1.711 (0.802–3.652)				1.692 (0.758–3.777)	
2	3.827 (1.749–8.373)				2.823 (1.212–6.577)	
*Treatment break*		0.260		-		-
No	1.000					
Yes	1.548 (0.724–3.309)					
*Grade ≥ 3 toxicity*		0.204		-		-
No	1.000					
Yes	0.653 (0.339–1.259)					

Note: HR: Hazard ratio; CI: Confidence interval.

### Prognostic impact of mGPS and HS-mGPS for OS

**Figure 1:**
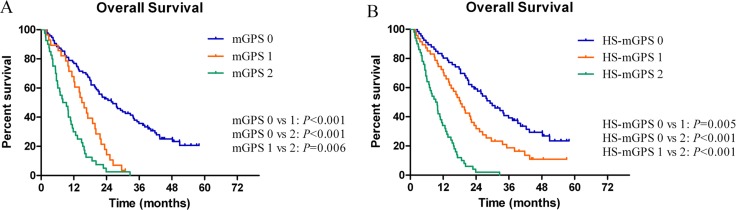
**(A, B)** Significant differences of overall survival according to the mGPS and HS-mGPS.

**Figure 2:**
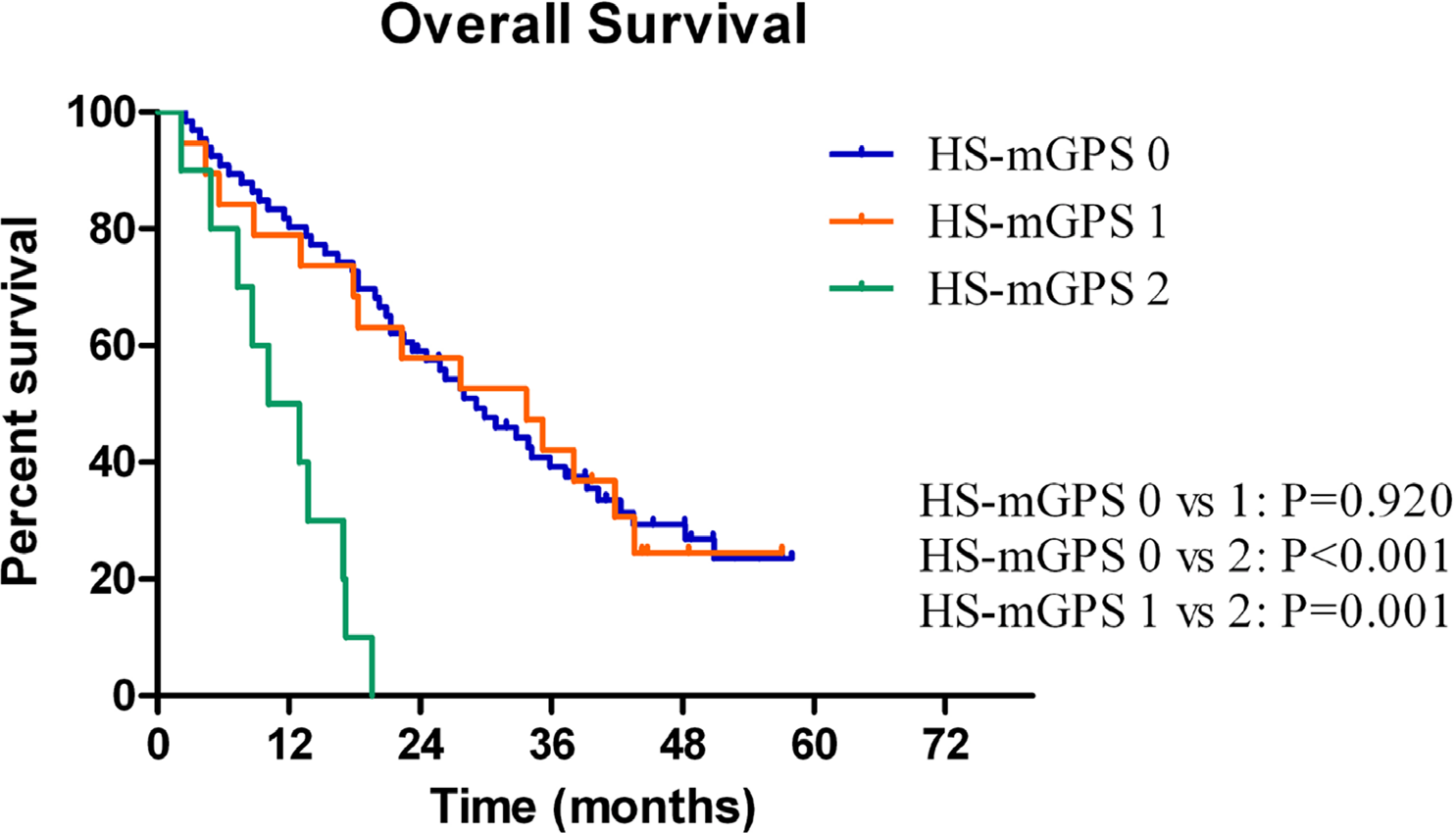
A significant difference was observed according to the HS-mGPS among the patients in the mGPS-0 group

The median OS time was 17.1 ± 1.4 months (95% CI: 14.4–19.9) for the whole population. The 1- and 3-year OS rates were 63.9% (95% CI: 0.565–0.713) and 20.9% (95% CI: 0.144–0.274), respectively. Kaplan-Meier survival analysis was then performed to evaluate the differences in prognostic impact between mGPS and HS-mGPS. Increases in mGPS were correlated with unfavorable OS (0 *vs.* 1, *P* < 0.001; 0 *vs.* 2, *P* < 0.001; 1 *vs.* 2, *P* = 0.006) (Figure 1A). In the same way, an elevated HS-mGPS was also significantly associated with reduced OS (0 *vs.* 1, *P* = 0.005; 0 *vs.* 2, *P* < 0.001; 1 *vs.* 2, *P* < 0.001; Figure 1B). In the subgroup analysis of mGPS0 group, 29 patients (19 in HS-mGPS 1 and 10 in HS-mGPS 2) were identified with a migrated high HS-mGPS. Survival analysis also demonstrated a significantly decreased OS among these patients (0 *vs.* 1, *P* = 0.920; 0 *vs.* 2, *P* < 0.001; 1 *vs.* 2, *P* = 0.001; Figure 2).

Univariate analysis identified that ECOG PS (*P* = 0.001), T stage (*P* < 0.001), N stage (*P* < 0.001), clinical stage (*P* < 0.001), tumor length (*P* = 0.004), BMI (*P* = 0.002), treatment modality (*P* < 0.001), mGPS (*P* < 0.001), HS-mGPS (*P* < 0.001) and tumor response (*P* < 0.001) were potentially prognostic factors for OS (Table 3). In the multivariate analysis, the factors associated with OS in the HS-mGPS model were: T stage ( *P* = 0.001; HR 1.889, 95% CI: 1.311–2.722), N stage (*P* = 0.030; HR 1.619, 95% CI: 1.047–2.505), clinical stage (*P* < 0.001; HR 2.693, 95% CI: 1.766–4.108), tumor response (*P* = 0.024; HR 1.549, 95% CI: 1.059–2.265) and HS-mGPS (*P* < 0.001; 0 *vs.* 1, HR 1.723, 95% CI: 1.104–2.690; 0 *vs.* 2, HR 3.727, 95% CI: 2.219–6.259). The corresponding figures in the mGPS model were: T stage (*P* = 0.004; HR 1.710, 95% CI: 1.192–2.452), N stage (*P* = 0.028; HR 1.612, 95% CI: 1.053–2.467), clinical stage (*P* < 0.001; HR 2.706, 95% CI: 1.793–4.085), tumor response (*P* = 0.004; HR 1.753, 95% CI: 1.198–2.566) and mGPS (*P* < 0.001; 0 *vs.* 1, HR 2.363, 95% CI: 1.453–3.841; 0 *vs.* 2, HR 2.908, 95% CI: 1.767–4.784) (Table 3). On Cox multivariate analysis which contained all prognostic factors, HS-mGPS showed a superior prognostic impact than the mGPS (*P* = 0.006; HR 1.677, 95% CI: 1.158–2.429; Supplementary Table 1).

**Table 3:** Univariate and multivariate analysis for OS

Variables	Univariate analysis		Multivariate analysis with mGPS		Multivariate analysis with HS-mGPS	
	HR (95% CI)	*P*-value	HR (95% CI)	*P*-value	HR (95% CI)	*P*-value
*Age (y)*		0.315		-		-
< 57	1.000					
≥ 57	0.841 (0.600–1.179)					
*Sex*		0.519		-		-
Male	1.000					
Female	0.860 (0.545–1.358)					
*ECOG PS*		0.001		0.090		0.068
0–1	1.000		1.000		1.000	
2	1.759 (1.244–2.487)		1.476 (0.942–2.314)		1.528 (0.969–2.411)	
*T stage*		< 0.001		***0.004***		***0.001***
T_3_	1.000		1.000		1.000	
T_4_	2.006 (1.427–2.820)		1.710 (1.192–2.452)		1.889 (1.311–2.722)	
*N stage*		< 0.001		***0.028***		***0.030***
N_0_	1.000		1.000		1.000	
N_1_	2.326 (1.579–3.428)		1.612 (1.053–2.467)		1.619 (1.047–2.505)	
*Clinical stage*		< 0.001		***< 0.001***		***< 0.001***
II-III	1.000		1.000		1.000	
IV_a_	3.785 (2.653–5.402)		2.706 (1.793–4.085)		2.693 (1.766–4.108)	
*Tumour length (cm)*		0.004		0.566		0.510
< 5	1.000		1.000		1.000	
≥ 5	1.676 (1.176–2.388)		1.118 (0.763–1.638)		1.143 (0.768–1.701)	
*Tumour location*		0.450		-		-
Upper	1.000					
Middle	0.796 (0.543–1.167)					
Lower	0.875 (0.564–1.358)					
*Differentiation*		0.507		-		-
Well	1.000					
Fairly	0.900 (0.585–1.384)					
Poorly	0.736 (0.498–1.087)					
*Weight loss (%)*		0.073		-		-
< 10	1.000					
≥ 10	1.376 (0.971–1.950)					
*BMI (Kg/m*^2^*)*		0.002		0.105		0.127
< 18.5	1.000		1.000		1.000	
18.5 ≤ BMI< 22.9	0.571 (0.382–0.854)		0.691 (0.433–1.100)		0.757 (0.475–1.204)	
≥ 23	0.448 (0.265–0.757)		0.594 (0.335–1.054)		0.636 (0.355–1.140)	
*RT delivery*		0.887		-		-
3D-CRT	1.000					
IMRT	1.026 (0.722–1.458)					
*Treatment modality*		< 0.001		0.373		0.277
TP	1.000		1.000		1.000	
PF	2.315 (1.621–3.304)		1.248 (0.767–2.032)		1.310 (0.806–2.129)	
*mGPS*		< 0.001		***< 0.001***		-
0	1.000		1.000			
1	2.683 (1.671–4.308)		2.363 (1.453–3.841)			
2	5.179 (3.344–8.022)		2.908 (1.767–4.784)			
*HS-mGPS*		< 0.001		-		< 0.001
0	1.000				1.000	
1	1.828 (1.195–2.796)				1.723 (1.104–2.690)	
2	5.769 (3.678–9.048)				3.727 (2.219–6.259)	
*Treatment break*		0.323		-		-
No	1.000					
Yes	1.230 (0.816–1.854)					
*Grade ≥3 toxicity*		0.943		-		-
No	1.000					
Yes	1.013 (0.708–1.450)					
*Tumour response*		<0.001		***0.004***		***0.024***
CR+PR	1.000		1.000		1.000	
SD+PD	2.278 (1.619–3.204)		1.753 (1.198–2.566)		1.549 (1.059–2.265)	

Note: HR: Hazard ratio; CI: Confidence interval.

## DISCUSSION

**Figure 3:**
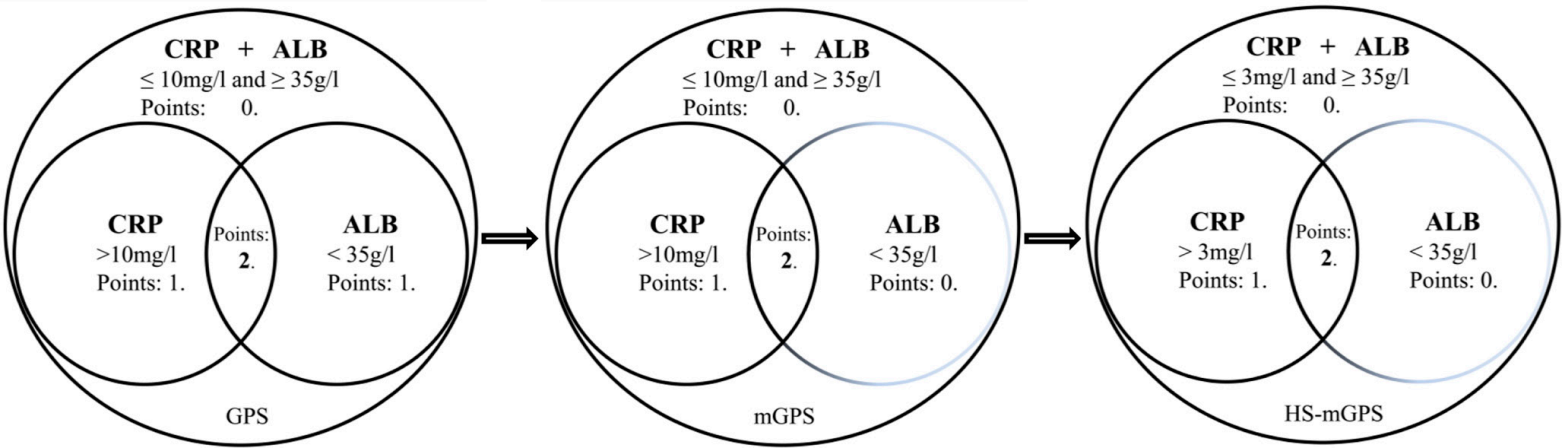
A brief evolution of the GPS and its derivatives

A major limitation of the present prognostic instruments, e.g. the Karnofsky performance status (KPS), the Eastern Cooperative Oncology Group (ECOG PS), and Palliative PS, is a reliance on subjective clinical measurements. Therefore, at the beginning of this century, Forrest *et al.* reported the first study, which combined routine objective markers of the systemic inflammatory response (CRP and ALB, termed as GPS), and showed superiority than the clinical standard combination of TNM system and ECOG PS on survivals in various cancer types [8–11]. Subsequent investigations further refined this inflammation-based prognostic system to mGPS and HS-mGPS (detailed in Figure 3). As demonstrated in this study, although both the mGPS (*P* < 0.001) and HS-mGPS (*P* < 0.001) were strong prognostic predictors for OS in LAESCC patients who received CCRT, only the HS-mGPS was a positive factor for tumor response (*P* = 0.015). In addition, the HS-mGPS was found to be a superior prognostic predictor compared to the mGPS for OS in multivariate analysis (*P* = 0.006).

Recently, there has been an increasing discussion about the prognostic values of GPS and its derivatives in esophageal cancer. Kimuria *et al.* reviewed 142 stages III-IV ESCC patients receiving CCRT based on PF regimen. ORR was documented in 84 patients (59.2%). In logistic regression analysis, they found that GPS was one of the independent predictors of response to CCRT (*P* = 0.002). Furthermore, GPS was also a strong prognostic indicator for disease-specific survival (*P* = 0.002) and progression-free survival (*P* = 0.002) in their study [12]. Zhang *et al.* evaluated the potential prognostic significance of the mGPS and another hotly investigated inflammation-based prognostic score titled NLR (neutrophil/lymphocyte ratio) in 212 inoperable ESCC patients who received CCRT [13, 14]. Their results showed that the 3-year OS rate for the entire cohort was 24.6% with the median OS time for all patients was 17.0 months, which was quite consistent with our results. In multivariate analysis, mGPS (*P* < 0.001; HR 1.694, 95% CI: 1.350–2.126) was found to be one of the three independent prognostic factors affecting OS and the other two predictors were T stage and M stage. Receiver operating characteristic (ROC) curve also verified that the predictive ability of the mGPS was superior to that of the NLR (*P* = 0.048).

Although Proctor *et al.* had already confirmed that the HS-mGPS could enhance the prognostic values of the GPS and mGPS in a large cohort of patients (*n* = 12,119) with cancer [4], there are very fewer data on the application of HS-mGPS in esophageal cancer patients who received CCRT. Of the 163 unresectable LAESCC patients in this study, both the higher scores of mGPS and HS-mGPS were significantly correlated with more progressive diseases and pretreatment malnutrition. It is interesting to note that, for the 95 patients in the mGPS-0 group, 29 patients migrated to the high score group according to the HS-mGPS criterion and due to this migration, the HS-mGPS could reflect the prognosis more sensitively than the mGPS (*P* = 0.006, Figure 2). Results of the current study are in agreement with those of previous reports. In a large sample retrospective study, 552 resectable gastric cancer patients were reviewed and compared the prognostic significance of the preoperative mGPS and HS-mGPS. Although both the mGPS and HS-mGPS were good prognosticators (both *P* < 0.001) for OS, the HS-mGPS was found to be a superior prognostic predictor compared to the mGPS in multivariate Cox regression analysis (*P* = 0.0002; HR 1.6748, 95% CI: 1.2867–2.1314) [5]. In another study which compared the prognostic value of the GPS, mGPS, HS-mGPS and other inflammation-based markers in 327 patients with resectable non-small cell lung cancer, results also supported that HS-mGPS (*P* = 0.016; HR 2.777, 95% CI: 1.210–6.374) was an independent prognostic factor than GPS and mGPS for OS [6]. Taken together, there is a possibility that HS-mGPS is a more suitable prognostic marker for LAESCC patients who received CCRT than mGPS.

A major limitation of the present study is its confinement to the retrospective design. Although we conducted this analysis in multiple cancer centers, some potential factors might have influences on the results. Large-scale prospective study is highly warranted in the future.

In conclusion, HS-mGPS is an objective, independent predictive factor for treatment response in LAESCC patients receiving CCRT and a better prognostic indicator for OS than mGPS.

## MATERIALS AND METHODS

### Study population

The present study was conducted between January 2011 and December 2014 at three endemic areas of ESCC in China (Department of Radiation Oncology, Hangzhou Cancer Hospital; Department of Radiation Oncology, Zhejiang Provincial People’s Hospital; Department of Radiation Oncology, The First Affiliated Hospital of Wenzhou Medical University). Written informed consents were obtained from all the patients and the Ethic Committees of all participating cancer centers approved for data analysis (Hangzhou Cancer Hospital, Zhejiang Provincial People’s Hospital and The First Affiliated Hospital of Wenzhou Medical University).

In total, 163 patients with cytopathologically confirmed primary ESCC were retrospectively collected. The exclusion criterias were as follows: I). early-stage esophageal cancer or adenocarcinoma of the esophagus; II). patients who received previous anti-inflammatory drugs within 1 week; III). incomplete data of pretreatment CRP and ALB.

### Pretreatment work-up

The pretreatment work-up included complete history collection, physical examination, electrocardiography, and blood tests (CRP concentrations were immunoturbidimetrically measured using a Roche clinical chemistry assay (Roche Diagnostics, Belleville, NJ, USA). ALB was quantified using automatic biochemical analyzer (Beckman Coulter AU5800, Beckman Coulter, Fullerton, CA, USA). The extent of disease evaluation included endoscopy of the esophagus, barium swallowing, computed tomography (CT) and positron emission tomography/CT (PET/CT, if available). Bone scans were performed if clinically indicated. Clinical stages (II–IVa) were diagnosed according to the 6th edition of the American Joint Committee on Cancer TNM staging system.

Blood test results within 1 week before radiotherapy were used as an evaluation of the mGPS and HS-mGPS. In addition, the measurements of CRP were repeated after 5–7 days if there were signs of infection including fever (> 38°C) or white blood cell count ≥ 10,000/mm^3^. The lowest serum CRP level was then used for analysis. The score of mGPS was defined as follows: patients with both an elevated CRP level (> 10 mg/l) and hypoalbuminemia ( < 35 mg/l) were allocated a score of 2; patients in whom with only an abnormal CRP level were given a score of 1 and those with a normal CRP level (≤ 10 mg/l) were given a score of 0. In terms of HS-mGPS, the cutoff value of CRP was decreased to 3 mg/l. In addition, a patient’s BMI was calculated and classified according to the Asian-specific BMI cutoff values as follows: underweight ( < 18.5 kg/m^2^); normal weight (18.5–22.9 kg/m^2^); overweight and obese (≥ 23.0 kg/m^2^) [15].

### Treatment protocol

114 patients (69.9%) received chemotherapy based on cisplatin and paclitaxel (TP regimen). Cisplatin at 75 mg/m^2^ was administered intravenously on Day 1 and Day 29 with standard hydration, followed by paclitaxel at 135 mg/m^2^ *i.v.* administered for 3 hours on the same days. The other 49 patients received two cycles of 5-Fu (250 mg/m^2^/day) on Days 1–4 and 29–32, and cisplatin (75 mg/m^2^) at day 1 of every 28-day cycle. In total, 106 patients (65.0%) received three-dimensional conformal radiotherapy (3D-CRT) and 57 patients were treated with intensity-modulated radiation therapy (IMRT). The preplanned radiation dose was 54.0–60.0 Gy, which was given as 30 fractions of 1.8–2.0 Gy once-daily fractions for 5 days per week. Treatment volumes (GTV, CTV and PTV) and dose-volume constraints of normal tissues have been described elsewhere [16]. Dose modification or suspension of treatment was considered if any grade 4 toxicities occurred and restarted when toxicities recovered to grades ≤ 2.

### Treatment assessment and follow-up

Clinical response was assessed according to the RECIST (Response Evaluation Criteria in Solid Tumors) system 4–6 weeks after the completion of treatment. The National Cancer Institute Common Toxicity Criteria (Version 4.0) was used to score acute treatment toxicity. Follow-up modalities included physical examination, blood tests, upper endoscopy, enhanced CT of the neck (mandatory for cervical esophageal cancer), chest, abdomen, and pelvis. Patients were followed up every 3 months for the first year, every 6 months for the second year, and then on a yearly basis.

### Statistical analysis

The cutoff date of the last follow-up was June 30, 2017 for the censored data analysis. χ^2^ test and Mann-Whitney *U* test were used to compare noncontinuous data as appropriate. OS was determined from the date of CCRT initiation to the last follow-up or to the date of death. Survival curves were generated using the Kaplan-Meier method and compared with the log-rank test. Tumor response was categorized as 1 (Responder: complete response [CR] and partial response [PR]) and 2 (Nonresponder: stable disease [SD] and progressive disease [PD]) for the purpose of analysis. A univariate analysis was performed to identify the predictive factors for the response to CCRT on one hand and to OS on the other hand. Variables identified with a 2-sided *P* value < 0.05 on univariate analysis were included in the multivariate analyses. Multivariate analysis of the predictive factors for the response to CCRT was performed using binary logistic regression with calculation of the hazard ratio (HR) and a 95% CI. Multivariate analysis of the predictive factors of OS was performed using a Cox regression model. A *P* value of < 0.05 was considered as statistically significant. All statistical analyses were conducted using IBM SPSS for Windows version 22.0 (SPSS, Armonk, New York, USA).

## SUPPLEMENTARY MATERIALS TABLE


