# Supplementary material for: High-sensitivity modified Glasgow prognostic score (HS-mGPS) Is superior to the mGPS in esophageal cancer patients treated with chemoradiotherapy
Source: Oncotarget. 2017 Oct 11;8(59):99861–70. doi: 10.18632/oncotarget.21734 (PMC5725136; doi:10.18632/oncotarget.21734)
Supplement: Supplementary file 1 [file oncotarget-08-99861-s001.pdf]

High-sensitivity modified glasgow prognostic score (HS-mGPS) Is superior to the mGPS in esophageal cancer patients treated with chemoradiotherapy

SUPPLEMENTARY MATERIALS

Supplementary Table 1: Multivariate analysis of all significant factors related to OS in LAESCC patients treated with CCRT

Factor	*p*-value	HR	95% CI
ECOG PS	0.085	1.491	0.946–2.348
T stage	***0.001***	1.839	1.275–2.652
N stage	***0.026***	1.632	1.062–2.507
Clinical stage	**< *0.001***	2.680	1.773–4.049
Tumour length	0.546	1.125	0.767–1.649
BMI	0.130	0.800	0.598–1.068
Treatment modality	0.269	1.314	0.810–2.132
Tumor response	***0.022***	1.562	1.068–2.285
mGPS	0.309	1.196	0.847–1.690
HS-mGPS	***0.006***	1.677	1.158–2.429

Note: HR: Hazard ratio; CI: Confidence interval.
